# Serum Albumin in Health and Disease: From Comparative Biochemistry to Translational Medicine

**DOI:** 10.3390/ijms241813725

**Published:** 2023-09-06

**Authors:** Daria A. Belinskaia, Richard O. Jenkins, Nikolay V. Goncharov

**Affiliations:** 1 Sechenov Institute of Evolutionary Physiology and Biochemistry, Russian Academy of Sciences, Thorez 44, St. Petersburg 194223, Russia; d_belinskaya@mail.ru; 2 Leicester School of Allied Health Sciences, De Montfort University, The Gateway, Leicester LE1 9BH, UK; richard.jenkins@dmu.ac.uk

Being one of the main proteins in the human body and many animal species, albumin plays a decisive role in the transport of various ions, electrically neutral molecules and in maintaining the colloid osmotic pressure of the blood. Albumin is able to bind almost all known pharmaceuticals, many nutraceuticals and toxic compounds, largely determining their pharmaco- and toxicokinetics [1,2]. However, albumin is not only a passive but also an active participant in pharmacokinetic and toxicokinetic processes, having a number of enzymatic activities [3]. Due to the free thiol group of Cys34, albumin can serve as a trap for reactive oxygen and nitrogen species, thus participating in redox processes [4]. The interaction of the protein with blood cells, blood vessels, as well as with parenchymal cells outside the vascular bed are also of great importance. Interaction of albumin with endothelial glycocalyx and vascular endothelial cells largely determines its integrative role [5]. Our Special Issue aimed to expand the current knowledge of serum albumin in both physiological and pathological conditions.

The analysis of the articles published in our Special Issue and other available literature has identified several key directions of the studies devoted to albumin. One of them is the participation of albumin in the pharmacokinetics of pharmaceuticals. Thus, Rodriguez-Alfonso et al. studied the pharmacokinetics of EPI-X4, an endogenous peptide inhibitor of CXCR4 (fusin, CD184, one of the chemokine receptors that the human immunodeficiency virus uses to infect CD4+ T-lymphocytes), which has therapeutic efficacy when applied topically in animal models of asthma and dermatitis [6]. The authors showed that EPI-X4 can covalently bind to human serum albumin (HSA), forming a disulfide bridge with the free thiol group of Cys34 of albumin, which increases the stability of the peptide in plasma. Therefore, pre-crosslinking of EPI-X4 with albumin in vitro prior to in vivo administration of the drug can significantly improve the pharmacokinetic properties of this new class of CXCR4 antagonists. Botti et al. investigated the interaction HSA and microRNA 155 (miR-155, regulates B cell differentiation, development stages, etc.) and showed that miR-155 can bind in one of the positively charged pockets of HSA [7]. Thus, native HSA can be used under physiological conditions for delivering miRNA to its molecular targets.

Sanaeifar et al. studied the interaction of spin-labeled naproxen derivative (SL-NPX) with bovine serum albumin (BSA) hydrogels [8]. It has been found that the rate of SL-NPX release from BSA hydrogels can be controlled by ethanol concentration, BSA concentration, incubation time, and initial drug loading percentage. These results lead to the conclusion that BSA hydrogels can be used in medicine for controlled drug delivery. Jagusiak et al. designed a model carrier system consisting of supramolecular albumin and Congo red [9]. The authors showed that this system can effectively bind the chemotherapeutic agent doxorubicin, while the drug can be controllably released at low pH. Thus, the system can become an alternative to the existing methods of targeted delivery of anticancer drugs to their molecular targets. In the frame of the studies on the pathogenesis of acute respiratory distress syndrome (ARDS), Kryvenko et al. investigated the mechanisms of cellular albumin uptake in a 3D culture of precision lung sections (PCLS) [10]. It was found that up to 60% of PCLS cells contained labeled albumin, while the absorption of albumin occurs by a receptor-mediated mechanism. The distribution of albumin in the cells indicated the transcytosis of the protein. The findings could help develop new ways to deliver drugs to the lungs, which could improve the chances of patients with respiratory failure to recover.

It is known that the albumin molecule is liable to allosteric modulation: the binding of one ligand in one binding site affects the binding of other ligands at another site. Targeted influence on albumin with the help of the molecules that modulate its functional properties could solve many problems in toxicology and pharmacology. For example, Litus et al. [11] showed that ibuprofen (IBU), an exogenous HSA ligand, increases the affinity of albumin for the monomer of β-amyloid peptide (Aβ, a key pathogenic factor in the development of Alzheimer’s disease). The revealed increase in HSA-Aβ interaction under the action of IBU (and as a result, enhanced inhibition of Aβ fibrillation under the action of HSA in the presence of IBU) may be an alternative way to prevent the deposition of β-amyloid peptide in the brain.

Another topical area of research on albumin is associated with the search for new diagnostic and prognostic indices. Albumin serves as a predictor of the outcome of many pathologies, being one of the main biomarkers in diagnosis. Albumin, being the main protein in blood plasma and other body fluids, takes the first blow under conditions of oxidative stress, so the redox status of albumin is widely used as a biomarker of various pathological conditions [4]. Paar et al. [12] investigated the redox status of HSA in patients with multiple sclerosis. The percentage of reduced and oxidized albumin in the serum and cerebrospinal fluid (CSF) of the patients and healthy subjects was analyzed. A trend towards an increased content of irreversibly oxidized HSA in CSF of the patients compared with the control was revealed. The degree of oxidation of serum albumin was associated with disability during remission, while the redox status of albumin in CSF was associated with disease activity.

In a study by Wybranowski et al. [13], 66 hospitalized COVID-19 patients with respiratory symptoms were inspected. It was found that inflammatory parameters and radiological scores of computed tomography (HRCT) significantly correlated with the level of albumin (negative correlation) and the level of advanced oxidation protein products (AOPPs, positive correlation). The ratio of AOPPs/albumin significantly correlated with the level of D-dimers. The revealed correlations confirm the involvement of oxidative stress in the pathogenesis of COVID-19. These findings broaden knowledge of the role of oxidized albumin in SARS-CoV-2 infection: oxidized albumin is not just a biomarker of oxidative stress; it may be involved in the pathophysiology of COVID-19.

Although the level of albumin is widely used as a diagnostic and prognostic marker, little is known about how the structural integrity and functional activity of albumin correlates with the pathophysiological state of patients. A pilot study by Tsao et al. [14] showed that serum albumin in 74-year-old subjects was more prone to misfolding compared to 24-year-old ones. The authors hypothesized that misfolding of serum albumin in the elderlies may be, at least in part, the result of age-related oxidative stress. Improperly folded albumin can lose its ability to realize its functional role and can become a pathophysiological link in various diseases. Wu et al. [15] performed a meta-analysis of seventeen clinical trials involving 5828 cancer patients to investigate whether the High-Sensitivity Modified Glasgow Prognostic Score (HS-mGPS), which takes into account albumin and C-reactive protein levels, can accurately predict the survival of cancer patients. It turned out that HS-mGPS can indeed serve as a reliable prognostic biomarker for cancer patients: a high level of HS-mGPS is associated with poor survival outcomes.

Within the framework of the directions described above, our long-term studies are devoted to the (pseudo)esterase activity of albumin and how this activity changes under the influence of various xenobiotics or/and pathological conditions [16,17,18,19]. The contribution of albumin to the esterase status of human blood plasma is of particular interest. Esterase status is the set, amount and ratio of the activity of the enzymes from the subclass of esterases [20]. Esterases play an important role in many key physiological processes in the body, so the esterase status of blood plasma can claim to be one of the universal markers of various infectious and non-infectious pathologies, including COVID-19. In our recent study [21], we compared the esterase status (taking into account the amount and enzymatic activity of HSA) and other blood biochemical parameters in three groups of patients: healthy elderly subjects, and surviving and deceased patients with confirmed COVID-19. Statistically significant differences in esterase status and biochemical parameters (including albumin level) were found between healthy individuals and patients with COVID-19, as well as between surviving and deceased patients. A new diagnostic marker [Urea] × [MDA] × 1000/(BChEb × [ALB]) was identified, which was 10 times higher in the group of deceased patients vs. the group of survivors, and 26 times higher than in the group of healthy elderly individuals. The results of our clinical biochemical tests provide grounds for further large-scale clinical trials in accordance with the requirements of evidence-based medicine.

It is interesting to note that every year more and more new biomarkers of the severity of various diseases appear, united by a common denominator—the level of albumin. It emphasizes the integrative role of this protein, which serves as a link between various tissues and organs. As an example, here are a few of these markers described in recent studies: neutrophil/pre-albumin for diagnosing and predicting disease activity in patients with inflammatory bowel disease [22]; lactate/albumin as a predictor of in-hospital mortality in hospitalized cirrhotics [23]; neutrophil/albumin and C-reactive protein/albumin as novel inflammatory markers in Behcet’s disease [24]; zinc/albumin as potential biomarkers for a precision medicine approach to COVID-19 [25]; ferritin/albumin as a new indicator of COVID-19 disease mortality [26]; blood urea nitrogen/albumin for predicting prognostic outcomes of inpatients with COVID-19 [27]; procalcitonin/albumin for identifying sepsis in neonates with pneumonia [28]; international normalized ratio/albumin as a novel marker of upper gastrointestinal bleeding severity [29].

Thus, modern diagnostics, pathogenesis of various diseases, and the development of therapeutic agents are currently virtually unthinkable without a comprehensive account of the physicochemical, evolutionary, genetic, and physiological and biochemical characteristics of albumin. However, albumin can serve not only as a biomarker of the severity of various pathologies, but also as a means of therapy. The study of this aspect is another important direction in research on albumin. Due to its essential physiological role, human albumin is the most sought after among other biopharmaceuticals. Thus, clinical indications for the use of a 4–5% solution of albumin (isooncotic solution) are hypovolemic shock, acute liver failure and cardiopulmonary bypass, while indications for the use of a 20–25% solution (hyperoncotic) are hypoalbuminemia, and sepsis or septic shock cirrhosis of the liver with ascites [30]. Yet it has not been practically studied what percentage of albumin should be in isotonic solutions of crystalloids during resuscitation in the case of hypoalbuminemia. The review by Wiedermann [31] published in our Special Issue summarizes the current evidence and conclusions drawn regarding the role of hypoalbuminemia in volume resuscitation. It was concluded that hypoalbuminemia acts as an effect moderator in volume resuscitation and plasma expansion with intravenous (IV) human albumin solutions. A review by Kuten Pella et al. [32] summarizes current approaches to the therapeutic use of albumin in regenerative medicine. Thus, albumin coating can be used to increase the biocompatibility of various types of implants. Albumin can be combined with natural or synthetic materials, resulting in novel composites suitable for nervous, cardiac system, and hard and soft tissue engineering. Several technologies described in the review have already entered the clinical phase.

Summing up, it can be noted that every year the ideas about the importance of albumin in human and animal organisms change. Once it was assigned the modest role of an osmotically active component, then the function of a transport protein was added to the list of albumin responsibilities, then it was revealed that the protein can be a universal biomarker of the severity of various pathologies. Now there is more and more evidence that the state of albumin (misfolding, oxidation, glycation) can determine human health, and the disruption of this protein is an independent pathogenetic factor in many diseases. Without a doubt, in the future, albumin will present us with many more surprises in the best sense of the word.

## Data Availability

Not applicable.
